# Island-wide population genomic analysis of the coastal shrub, beach naupaka (*Scaevola taccada* L.), on Oʻahu

**DOI:** 10.1093/aobpla/plag042

**Published:** 2026-09-04

**Authors:** Edward V McAssey, Erika R Moore-Pollard, Cassidy Downs, Karolina Heyduk

**Affiliations:** Department of Ecology and Evolutionary Biology, University of Connecticut, 75 North Eagleville Road, Storrs, CT 06269, United States; School of Life Sciences, University of Hawaiʻi at Mānoa, 3190 Maile Way, Honolulu, HI 96822, United States; Department of Ecology and Evolutionary Biology, University of Connecticut, 75 North Eagleville Road, Storrs, CT 06269, United States; School of Life Sciences, University of Hawaiʻi at Mānoa, 3190 Maile Way, Honolulu, HI 96822, United States; Department of Ecology and Evolutionary Biology, University of Connecticut, 75 North Eagleville Road, Storrs, CT 06269, United States; School of Life Sciences, University of Hawaiʻi at Mānoa, 3190 Maile Way, Honolulu, HI 96822, United States

**Keywords:** genetic diversity, Hawaiʻi, human influence, island, population genomics, population structure, Scaevola

## Abstract

Plant species face myriad threats to their survival, including climate change and habitat destruction. On Oʻahu in Hawaiʻi, *Scaevola taccada* L., a coastal dune shrub, is highly impacted by human activities and environmental threats. Despite its ubiquity across Pacific islands, the extent and distribution of genetic diversity within individual islands is unclear. We surveyed genomic diversity using double digest restriction-associated DNA sequencing (ddRAD-seq) in eight populations of *S. taccada* on Oʻahu. Using principal components (PCA) and STRUCTURE analyses, we assessed whether genetic diversity was structured geographically and the extent to which selection and migration structured genetic diversity. We analysed climate and leaf thickness data to understand how patterns of genetic diversity were correlated with environmental and trait variation. Our analyses pointed to a trend in which populations in protected areas were genetically differentiated from, and harboured less genetic diversity than, human-dominated areas. Analyses of genetic structure indicated that western beach populations were differentiated from eastern beach populations. *F*_ST_ outlier analyses suggest that selection is not primarily responsible for the differentiation seen between the protected populations and the human-dominated areas. Our results demonstrate that *S. taccada* populations in protected natural areas harboured unique genetic variation compared to human-dominated beach parks, but that protected populations also had significantly reduced genetic variation compared to most other populations. Relatively few *F*_ST_ outliers, paired with evidence of continued gene flow, suggest that current levels of genetic diversity are more a function of isolation and drift as opposed to selection.

## Teaser

Beach naupaka, Scaevola taccada, populations exist in protected and recreational coastal environments on Oʻahu, Hawaiʻi. We showed that even on a single island there is population genomic structure whereby protected areas are somewhat distinct from recreational areas. There was also a general trend in which those protected populations contained less genetic diversity compared to most of the recreational populations.

## Introduction

Islands are an ideal environment to study the distribution of genetic diversity in plants due to their isolation and relatively small size. Although small, islands contain a greater concentration of endemic species compared to continents (Kier et al. 2009) and contain many species of conservation concern (Schrader et al. 2024). For example, *Sabal lougheediana* M.P.Griff & Coolen is a single-island endemic found only on Bonaire in the Leeward Antilles. Sampling wild populations revealed low genetic diversity in one population (*H*_O_ = 0.019) and another population with only a single individual due to recent increases in residential buildings in the area (Clugston et al. 2024). While small population sizes are often correlated with reduced genetic diversity, the Corsican snail *Tyrrhenaria ceratina* maintains much higher genetic diversity (*H*_O_ = 0.532) in some populations and has significant genetic structuring, despite only being found in two hectares (Camus et al. 2024). In this case, conservation practices are credited with maintaining *T. ceratina* despite the small geographic range as the area has been fragmented by human activities over the years like the construction of an airport, parking lots, and urbanized development (Camus et al. 2024). Genomic techniques are further enhancing our ability to quantify the amount and distribution of genetic variation in island species. For example, *Kokia kauaiensis* (Rock) O.Deg. & Duvel is a single-island endemic species found in Kauiʻi in Hawaiʻi where whole genome resequencing was used to comprehensively sample wild populations to quantify relatively low genetic diversity but significant genetic structure (Ning et al. 2026). While many single-island studies of genetic diversity are necessarily focused on species of conservation concern, studying non-threatened taxa on single islands (as opposed to their full range) provides an important baseline that can be used to compare to other species. Furthermore, species with wide distributions like wild populations of *Daucus carota* L. have been studied on single islands, like Nantucket, MA, USA, to answer specific questions about the strength of crop-wild gene flow in the area (Mandel et al. 2016).

There are few islands as notable for their endemism as the Hawaiian Islands, where the most recent estimates suggest that of the approximately 1000 plant species found there, the vast majority (90%) are endemic (‘DLNR Rare Plant Program Website’). Plant species in Hawaiʻi have filled many available habitat types including coastal dunes, lava beds, cliffs, and sub-tropical rainforests. Orographic rain, caused by moisture moving up mountainous terrain, results in sharp environmental gradients in terms of both precipitation and temperature. The habitat variation across Hawai’i has set the stage for several adaptive plant radiations including in *Bidens* L. (Helenurm and Ganders 1985, Knope et al. 2020), *Schiedea* Cham. & Schltdl. (Kapralov and Filatov 2006), *Plantago* L. (Dunbar-Co et al. 2008), the silversword alliance (Witter and Carr 1988), and the lobeliads (Givnish et al. 2009).

**Figure 1:**
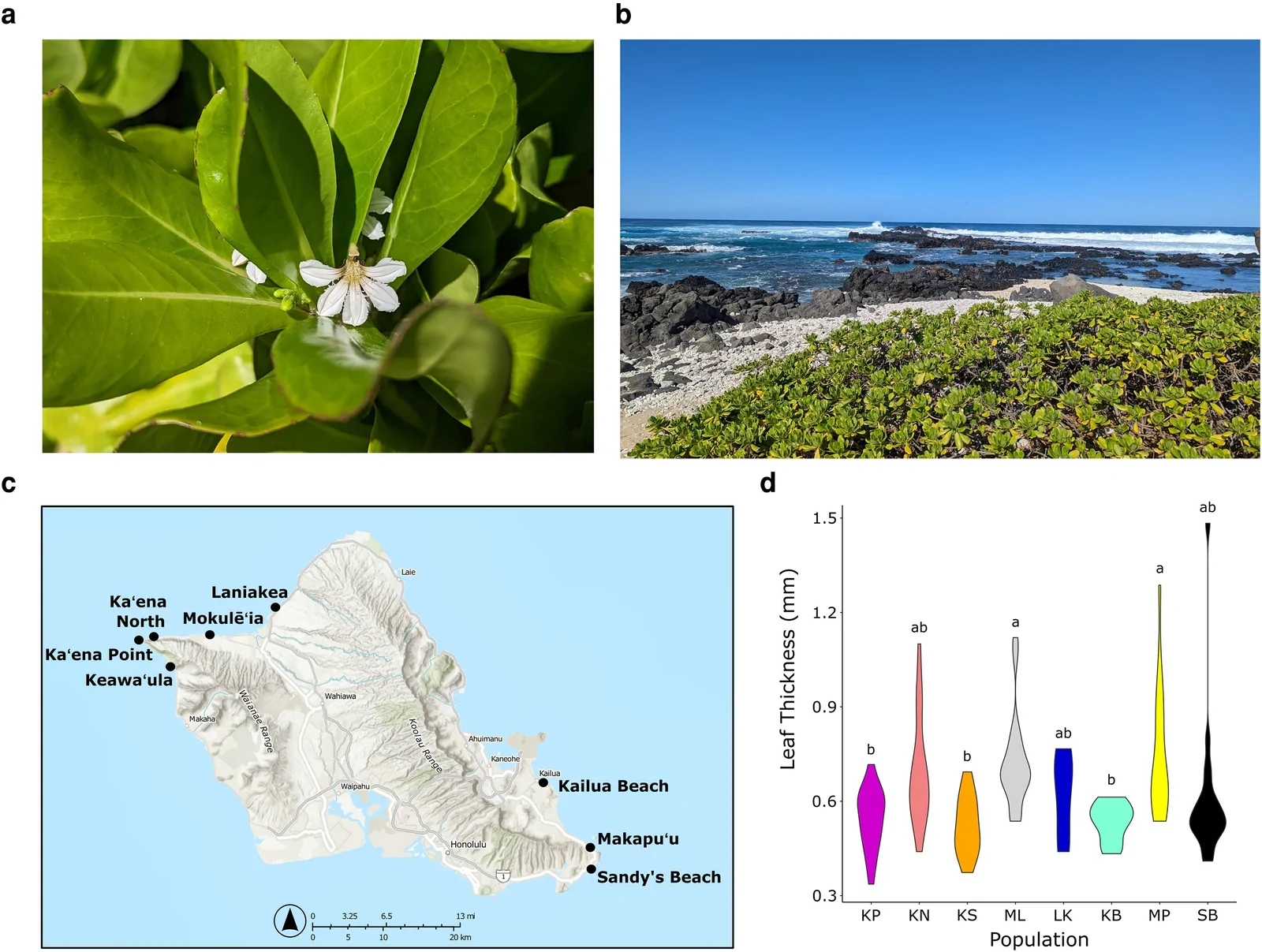
Species and habitat overview and leaf thickness population variation in *S. taccada*. (a) Flower of *S. taccada*; (b) Example dune habitat where *S. taccada* is found (Kaʻena Point); (c) Dots correspond to sampling locations on Oʻahu; Map made on Online ArcGIS (Using Esri, NASA, NGA, USGS, TomTom, Garmin, SafeGraph, FAO, METI, EPA, USFWS data); (d) Violin plot of leaf thickness variation in each population measured in millimetres; two letter population codes correspond to sampling locations found on the map in panel (c): KN—Kaʻena North; KP—Kaʻena Point; KS—Keawaʻula section of Kaʻena Point; KB—Kailua Beach; LK—Laniakea; MP—Makapuʻu; ML—Mokulēʻia; SB—Sandy’s Beach.

Other genera in Hawaiʻi, such as *Scaevola* L., inhabit a diverse set of habitats while not undergoing a large adaptive radiation (McKown et al. 2016). *Scaevola* arrived in Hawai’i via at least three independent dispersal events (Howarth et al. 2003). Two of the three dispersals have not undergone cladogenesis, whereas the third dispersal event resulted in seven endemic species, some of which are homoploid hybrids (Howarth and Baum 2005). The nine species of *Scaevola* in Hawaiʻi (naupaka in ʻŌlelo Hawaiʻi, Goodeniaceae) span significant environmental gradients in terms of precipitation and elevation that generally correlate with functional traits (McKown et al. 2016). In particular, leaf traits like leaf area, succulence, stomatal density, and vein density correlate with the environment when comparing all Hawaiian *Scaevola*. Morphological differences between species have long been recognized before colonial documentation of species on the islands. The Hawaiian name for *Scaevola*, Naupaka, derives from Hawaiian legend, after the princess Naupaka who fell in love with a fisherman (Nishimura 2016). Their love was forbidden by elders and by the gods, and so Naupaka tore a flower in half and sent the fisherman back to the ocean. Naupaka retreated back into the mountains with her half of the flower. The half-flower shape of *Scaevola* species (Fig. 1a) is representative of the two lovers torn apart forever; the legend is also emblematic of the multiple mountain species (naupaka kuahiwi) and the common coastal shrub *Scaevola taccada* Gaertn. (naupaka kahakai) (Wagner et al. 1999).

Unlike the *Scaevola* dispersal that produced seven species, *S. taccada* is a widespread coastal dune species in the Pacific basin and has not speciated after reaching Hawaiʻi (Howarth et al. 2003). The fruits of *S. taccada* are capable of floating for at least 180 days (Emura et al. 2014), which promotes its pan-Pacific distribution. However, some populations at higher elevations in south Japan have a higher proportion of pulp, which was hypothesized to facilitate dispersal by birds (Emura et al. 2014). While *S. taccada* is a common and abundant species across the greater Pacific basin, it is also an important species of dune ecosystems found on densely human populated islands such as Oʻahu Hawaiʻi (Fig. 1b) (Richmond and Mueller-Dombois 1972), providing habitat for notable species like the Laysan Albatross (*Phoebastria immutabilis*) (Young et al. 2009) and the Hawaiian Yellow-Faced Bee (*Hylaeus anthracinus*) (Graham et al. 2021). Dune ecosystems across the globe are facing threats from climate change, including sea level rise. An analysis of multiple mangrove plant species in southeast Asia suggests genetic diversity is being significantly reduced after decades of sea level rise in the region. Despite being adapted to wet environments, multiple mangrove species exhibit low effective population sizes, which is already having an impact on mollusk biodiversity in the area (Guo et al. 2018). In addition to providing habitat for other species, these coastal plant species provide essential buffering against extreme weather events and help hold soil in place (Sigren et al. 2014).

Recently, a genome assembly for an accession of *S. taccada* collected in Dongzhai Harbor, Hainan province, China, was published to enable evolutionary studies in the genus (He et al. 2022, Li et al. 2023). Separately, ddRAD-seq population genomic analyses were conducted with specimens collected from islands in south Japan to determine levels of gene flow and how gene flow relates to dimorphism in fruit type (cork vs. pulp) (Emura et al. 2022). Genomic analyses indicated that beach *S. taccada* populations exhibited relatively more admixture, presumably due to the relative ease of sea-based dispersal among beach populations, compared to higher elevation populations (Emura et al. 2022). A range-wide study of population genetic variation, using nuclear and plastid microsatellite markers (Banerjee et al. 2022), demonstrated that *S. taccada* originated in Australia before dispersing throughout its contemporary range. This species-wide study precluded more fine scale questions like the introductions to individual islands. For example, only a single accession was sampled from Oʻahu (Banerjee et al. 2022), which means that the precise levels and distribution of genetic diversity within this populous island in the Pacific remain undescribed.

Our goal in this study was to use genomic data to understand fine scale patterns of population genetic diversity and structure of *S. taccada* within Oʻahu, the third largest island of Hawaiʻi (approximately 1545 km^2^). Oʻahu represents a blend of natural and human-dominated environments and *S. taccada* is readily found in both. Relatively few island-wide population genomic studies have occurred on plants on Oʻahu although there have been previous population genetic studies in comparable study locations by DeJoode and Wendel (1992) and Cole and Morden (2021). Thus, we sought to determine whether the levels and structure of genetic diversity in natural populations on Oʻahu exhibited significant differences associated with geography and/or human use. Additionally, we surveyed leaf thickness across populations to determine whether variation in thickness is associated with differences in environmental conditions among populations.

## Materials and methods

### Sampling

*Scaevola taccada* populations were sampled across the island of Oʻahu (Fig. 1c) in August and September 2022. Populations were chosen to maximize the likelihood that they represent natural environments, as opposed to areas that are intermixed with housing/resorts that may be planting *S. taccada* as an ornamental plant. In total, we sampled eight representative populations: three in east Oʻahu and five in west Oʻahu. These populations varied in terms of human use: six populations within beach parks for recreation (Keawaʻula section of Kaʻena Point (KS), Kailua Beach (KB), Laniakea (LK), Makapuʻu (MP), Mokulēʻia (ML), Sandy’s (SB)) and two populations within a state park that is used for fishing and hiking (Kaʻena North (KN) and Kaʻena Point (KP)).

Due to *S. taccada’s* ability to reproduce vegetatively, within each population we sampled plants that were at least approximately 10 m from one another. Populations varied in terms of the number of plants that we could sample while maintaining a 10-m buffer between individuals. Population sample sizes ranged from six in Laniakea up to 20 individuals each in Kaʻena North, Mokulēʻia, and Sandy’s Beach with an overall average sampling per population of 15 individuals (Table 1). For each plant, young leaf tissue was sampled into a 15 ml conical tube and stored on ice. Additionally, to explore potential leaf water storage variation, we used digital callipers to measure leaf thickness for the three expanded and mature leaves per plant and averaged the values. Leaf thickness measurements were taken at the widest point of the leaf, and care was taken to avoid major veins. The GPS coordinates for all plants were recorded and an herbarium voucher specimen was deposited in the Joseph F. Rock Herbarium at the University of Hawaiʻi at Mānoa for each of the eight populations (Table 1). Tissue samples were stored in a −20°C freezer until processing.

**Table 1:** Geographic and environmental sampling information for *S. taccada* populations on Oʻahu.

Population	*N*	Latitude	Longitude	Average annual rainfall (mm)	Average driest month rainfall (mm)	Herbarium ID
Kaʻena North (KN)	20	21° 34.773′ N	158° 14.274′ W	768.8	22.3	HAW46034
Kaʻena Point (KP)	17	21° 34.497′ N	158° 16.44′ W	648.9	24.8	HAW46273
Keawaʻula (KS)	9	21° 33.187′ N	158° 14.777′ W	833.6	30.4	HAW46026
Kailua Beach (KB)	9	21° 23.902′ N	157° 43.741′ W	893.2	31.5	HAW46212
Laniakea (LK)	5	21° 37.091′ N	158° 5.145′ W	926.4	43.9	HAW46211
Makapuʻu (MP)	15	21° 18.980′ N	157° 39.835′ W	708.7	12.8	HAW46018
Mokulēʻia (ML)	19	21° 34.397′ N	158° 11.65′ W	829.8	16.7	HAW46016
Sandy’s Beach (SB)	20	21° 17.148′ N	157° 40.307′ W	698.6	12.7	HAW46021

### Phenotypic and environmental analyses

Leaf thickness measurements were averaged for each individual within a population. Population averages were compared via an ANOVA in R (version 4.4.1) and population statistical differences were detected with a Tukey’s HSD test with 95% confidence intervals (HSD.test within R package agricolae v1.3-7). Average yearly precipitation data and precipitation during the driest months were gathered from an established database (Giambelluca et al. 2013) at the University of Hawaiʻi that cover precipitation patterns from 1978 to 2007. Average yearly and driest month precipitation was correlated against average leaf thickness across populations to determine the extent that environmental variation played a role in establishing trait variation.

### DNA extraction and library construction

Frozen leaf tissue was ground in liquid nitrogen with a mortar and pestle, and DNA was isolated with a modified CTAB DNA extraction (Doyle and Doyle 1987, McAssey et al. 2023). Isolated DNA was quantified using the dsDNA BR Qubit Assay (Invitrogen, Waltham, Massachusetts, USA). DNA was submitted to the Center for Genome Innovation at the University of Connecticut for double digest restriction-associated DNA sequencing (ddRAD-seq) library preparation (Peterson et al. 2012). Samples were digested with *Msp*I and *Pst*I restriction enzymes and then ligated with barcoded adaptors. The *Msp*I adaptor contained a 10 basepair (bp) universal molecular indicator (UMI) to help identify and resolve PCR duplicates. Ligated products were amplified using Phusion MM (Thermo Fisher Scientific, Waltham, Massachusetts) with the following reaction conditions: 98°C for 30 s, 11 cycles of 98°C for 10 s, 65°C for 30 s, and 72°C for 30 s, followed by 5 minutes at 72°C. PCR products were 1× bead cleaned, assessed for quality on an Agilent Tapestation D1000 high-sensitivity assay, and sequenced on a portion of two separate Illumina NovaSeq 6000 lanes with 100 bp paired reads. De-duplicated and demultiplexed sequencing reads are available on NCBI’s Short Read Archive under BioProject PRJNA1268958.

### Sequence and population genetic analyses

Stacks v2.64 was used to process and analyse sequence data (Catchen et al. 2013). Within Stacks, the clone_filter script was used to remove PCR clones in our data by looking for exact sequences in paired end reads, which contained the 10 bp UMI sequence described above. Following clone filtering, Trimmomatic v0.39 was used to remove the UMI sequence, the first 10 bp of read two (Bolger et al. 2014). The process_radtags script in Stacks was used to demultiplex reads by barcode and to truncate the ends of read one to 90 basepairs to match the trimmed length of read two. Reads were then mapped to *S. taccada* genome (Li et al. 2023) using bwa mem v0.7.5a (Li and Durbin 2009). We retained reads with a MAPQ score greater than or equal to 30 using samtools v1.9 (Li and Durbin 2009). Within Stacks, the ref_map script was used to call single nucleotide polymorphisms (SNPs) from the genome mapped and filtered reads.

The flag -write_single_snp was used in Populations to ensure that no linked variants within a stack were analysed as they are not independent observations. A minor allele frequency (MAF) of 0.05, a maximum observed heterozygosity threshold of 0.7, and a minimum genotype read depth of four (−min-gt-depth 4 in Stacks v2.68) were all used to retain informative sites. Within Stacks we estimated population genetic parameters like percent polymorphic loci, observed heterozygosity, expected heterozygosity, and population differentiation (*F*_ST_). To determine whether the protected populations at Kaʻena Point had significantly different levels of genetic diversity than other populations on Oʻahu we performed a series of t-tests against the seven other populations. STRUCTURE v2.3.4 was used to further examine the existence of population structure within Oʻahu (Pritchard et al. 2000). Specifically, we used the admixture model with *K* ranging from 1 to 9. For each value of *K*, we ran 20 replicates with each replicate run consisting of 100 000 burn in and 1 000 000 MCMC iterations. The delta *K* method (Evanno et al. 2005) was implemented in STRUCTURE Harvester vB.1 (Earl and von Holdt 2012) to determine the most likely group of genetic clusters in the dataset. CLUMPP v1.1.2 (Jakobsson and Rosenberg 2007) and DISTRUCT v1.1 (Rosenberg 2004) were used to visualize STRUCTURE results. We visualized STRUCTURE results for the chosen *K* value (2) as well as additional *K* values (3 and 4) to determine the extent to any further sub-structure in our dataset. To further verify spatial genetic structure, we performed a principal components analysis (PCA) using the same polymorphisms analysed in the STRUCTURE analysis above using the R package SNPRelate (Zheng et al. 2012). To completement the STRUCTURE and PCA results we calculated a pairwise *F*_ST_ matrix using StAMPP (Pembleton et al. 2013) and tested for significance by performing 1000 bootstrap replicates and using a Benjamini-Hochberg multiple test correction (Benjamini and Hochberg 1995).

### Estimating migration events

As these populations were spread out over the island and we did not have extensive pollinator knowledge, we used the genetic data to assess patterns of gene flow. To detect potential gene flow events between populations using the filtered SNP dataset, we used TreeMix v1.13 (Pickrell and Pritchard 2012) to test for the existence of one to eight migration (m) events, given the total number of populations in our dataset. We then tested ten iterations of each number of m events and performed bootstrapping. Given the relatively low number of SNPs in our dataset (923 SNPs), we grouped SNPs in 75, 100, and 125 bp blocks using the -k flag (e.g. -k 100). We then determined the optimal number of m events for each block by evaluating iterations using the default settings of OptM (Fitak 2021). The m value with the highest likelihood value for each block, as determined by OptM, was then plotted using the R script provided by TreeMix, plotting_funcs.R.

### Effective population sizes

As these populations varied in their census population size, we sought to determine whether the effective population size (*N*_e_) also varied or if it was related to a population’s location. Our population genomic dataset provides an opportunity to use analyses of non-random associations between alleles to estimate *N*_e_ and its 95% confidence intervals. Effective population size (*N*_e_) was calculated for each population using the dartR (Gruber et al. 2018, Mijangos et al. 2022) package in R. First, a filtered vcf file containing the SNPs was converted into a GenLight object with population-level data using the function ‘gl.read.vcf’. Then, *N*_e_ was estimated using the Linkage Disequilibrium method via the ‘gl.LDNe’ function, with MAF set to 0.05, singleton alleles removed, and allowing random mating. The ‘gl.LDNe’ function requires NeEstimator v2.0.1 (Do et al. 2014), which was downloaded from GitHub (https://github.com/bunop/NeEstimator2.X).

### Geographic barriers to gene flow

While seed dispersal in *S. taccada* is assumed to be via ocean currents, we used genetic differentiation patterns to determine whether geography could explain which populations are more connected presumably via pollinator-mediated gene flow. We used pairwise *F*_ST_ and geographic locations of each population to assess whether there was any evidence of isolation by distance (IBD) in our data. IBD was assessed using Mantel tests between pairwise geographic distances and genetic distances. Statistical significance was evaluated using the ‘mantel’ function in vegan (Oksanen et al. 2026) with 9999 permutations.

### Selection

To understand the relative contribution that selection plays in population differentiation, we performed an *F*_ST_ outlier analysis using BayeScan (version 2.1; Foll and Gaggiotti 2008) to detect loci with high genetic differentiation consistent with local adaptation. We only used our putatively unlinked SNPs (from the write_single_snp filtering step within Stacks) in five separate runs of BayeScan using the default parameters. We further considered outlier loci that were detected in all five BayeScan runs to focus on the strongest candidates for local adaptation. We then used samtools to extract 500 bp upstream and downstream of each SNP to use in a BLAST search (Altschul et al. 1990) against the NCBI nucleotide database of Asterales to determine the extent to which outlier polymorphisms are in, or near, genes.

## Results

### Phenotypic and environmental variation

In a species that is capable of clonal reproduction, we first sought to determine the extent of phenotypic variation in the form of leaf thickness. Populations had significant variation in leaf thickness (*F*_7118_ = 4.949, *P* < 0.001), ranging from an average thickness of 0.52 mm at Keawaʻula to 0.75 mm at Makapuʻu (Fig. 1d). Populations exhibited very consistent average monthly temperatures for the warmest month of August ranging from 25.41°C to 25.76°C. The pattern of annual and summer precipitation was much less constant: average annual precipitation ranged between 648.9 mm at Kaʻena Point and 926.4 mm at Laniakea (Table 1). However, there was no discernible correlation between leaf thickness and annual precipitation. In contrast, when we correlated leaf thickness against the average monthly precipitation for the driest month, we detected a negative, but non-significant, trend (*R*^2^ = 0.275, *P* = 0.182; Supplementary Appendix S1). Unfortunately, the use of only eight populations in this study limits the statistical power to dissect this trend further via correlation-based approaches.

### Population genetic diversity

The average number of quality filtered paired reads per individual was 1 389 948 and ranged from 90 567 to 10 305 855. The average number of loci assembled per individual after mapping and filtering was 10 106 and ranged from 4246 to 18 689. The average coverage for these loci was 44.67× and ranged from 5.83× to 231.52×. Ultimately, 47 884 loci were genotyped in at least one individual in our dataset. To focus our genetic analysis on shared sites, we required each population to have 50% data present in order to include a locus in the analysis, resulting in 5357 loci that were analysed further. The average percent missing loci per individual was 5.63% and ranged from nearly no missing data (0.09%) to a few individuals with higher rates of missing data (three individuals with >20%; max missing data within an individual was 26.69%). Of the 5357 filtered loci, 923 contained SNPs used for population genetic analyses. The average number of individuals genotyped per population in our dataset was 13.48 (Table 2). For the 923 analysed SNPs, within population percent polymorphic loci ranged from 78.81% to 83.95% (Table 2). Populations exhibited variation in observed heterozygosity with an average of 0.276 and a range from 0.231 at Kaʻena Point to 0.336 at Laniakea (Table 2). To test whether Kaʻena Point had significantly lower observed heterozygosity than the other populations, we ran a series of *t*-tests in R. Kaʻena Point had the same level of observed heterozygosity compared to Kaʻena North (*P* = 0.238) and Makapuʻu (*P* = 0.092). However, Kaʻena Point had significantly lower observed heterozygosity than the remaining five populations (all tests *P* < 0.001). There were also differences in the average expected heterozygosity per population with an average of 0.262 and range from 0.221 at Kaʻena Point to 0.281 at Laniakea (Table 2). While some populations had *F*_IS_ values that were significantly different than 0 (Table 2), the differences were modest and did not reveal any trend regarding inbreeding.

**Table 2:** Within population estimates of genetic diversity.

Population	*N*	*P*	*H*_O_	*H*_E_	*F*_IS_
Kaʻena North (KN)	19.27	82.26	0.242 (0.006)	0.246 (0.006)	0.036 (0.053)*
Kaʻena Point (KP)	16.20	83.95	0.231 (0.007)	0.221 (0.006)	0.009 (0.050)
Keawaʻula (KS)	8.20	80.18	0.297 (0.008)	0.271 (0.006)	−0.013 (0.038)
Kailua (KB)	8.54	79.85	0.271 (0.007)	0.277 (0.006)	0.057 (0.030)*
Laniakea (LK)	4.81	78.81	0.336 (0.009)	0.281 (0.006)	−0.041 (0.014)*
Makapuʻu (MP)	14.03	81.54	0.247 (0.007)	0.252 (0.006)	0.047 (0.048)*
Mokuluēʻia (ML)	17.60	80.03	0.305 (0.007)	0.274 (0.006)	−0.052 (0.076)*
Sandy's Beach (SB)	19.19	79.94	0.280 (0.006)	0.276 (0.005)	0.007 (0.053)
Average	13.48	80.82	0.276	0.262	0.006

Two letter abbreviations correspond to Table 1 and Fig. 1. *N* = average number of individuals genotyped per population; *P* = percent polymorphic loci; *H*_O_ = observed heterozygosity; *H*_E_ = expected heterozygosity; *F*_IS_ = inbreeding coefficient. Numbers within parentheses represent the standard error. * indicates whether a population had an *F*_IS_ value significantly (*P* < 0.001) different than 0.

### Population genetic structure

**Figure 2:**
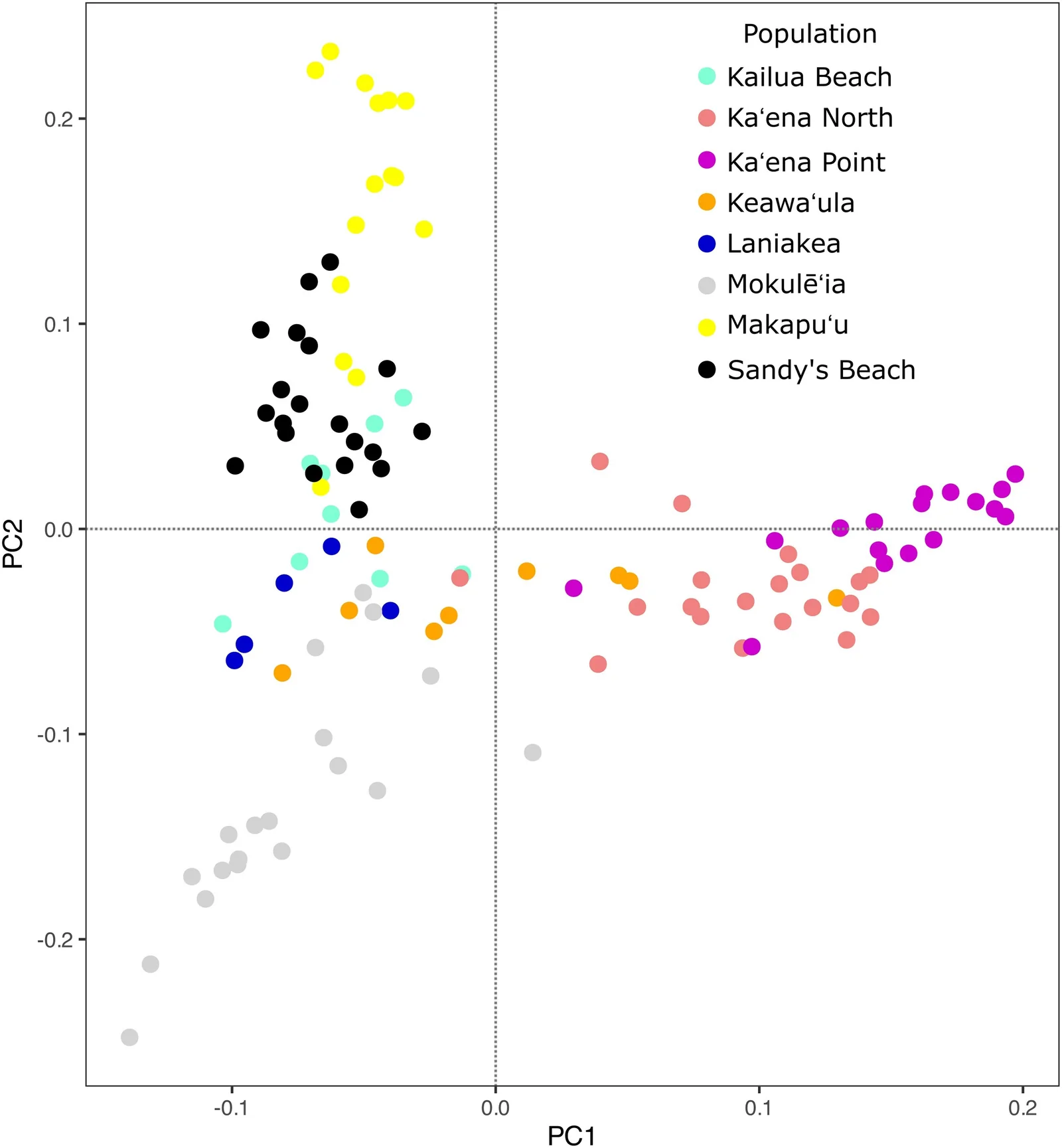
Principal components analysis plot based on 923 filtered SNPs showing the first two principal component axes. PC1 primarily separates protected populations (positive values on PC1) from non-protected populations and explains 10.74% of the variation in the 923 SNP dataset. PC2 explains 5.81% of the variation and generally separates populations geographically with eastern populations having positive values on PC2. Two letter population codes refer to the population labelling in Fig. 1 and Table 1.

**Figure 3:**
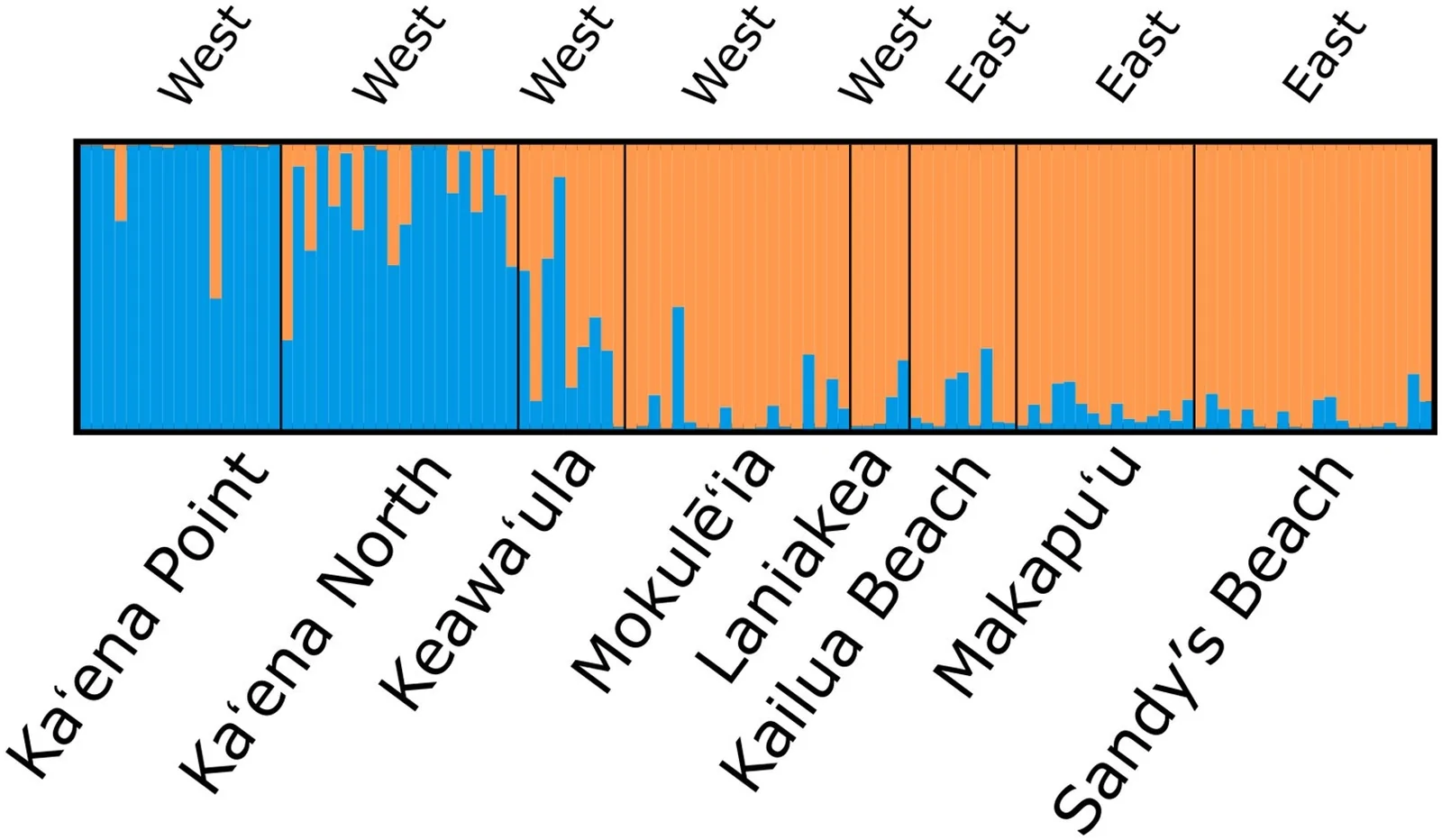
STRUCTURE plot based on 923 filtered SNPs. The delta-*K* method of Evanno et al. (2005) indicated *K* = 2 as being the most likely number of clusters in our dataset. East and West labelling refers to the population’s position on Oʻahu.

We then analysed whether populations around the island exhibited any level of population differentiation related to geographic location or human activity. Population pairwise *F*_ST_ values ranged from 0.024 to 0.208 with an average pairwise *F*_ST_ of 0.114 (Table 3). The principal component analysis (PCA) of the genotype data identified distinct grouping along PC1, which explains 10.74% of the variance in the dataset. Specifically, the Kaʻena North and Kaʻena Point populations have positive values on PC1 relative to the rest of the dataset (Fig. 2). PC2, which explains 5.81% of the variance, is generally separating out the non-Kaʻena North and Kaʻena Point populations by geography. Specifically, negative values on PC2 represent populations on west Oʻahu while positive values are east Oʻahu populations (Fig. 2). The PCA results largely correspond to the pairwise *F*_ST_ results. Both Kaʻena North and Kaʻena Point have consistently greater than average pairwise *F*_ST_ values when compared to other populations in this dataset. To explore this, we removed Kaʻena North and Kaʻena Point from the pairwise *F*_ST_ analysis and found that the average *F*_ST_ went down to 0.078, suggesting that those populations are driving the *F*_ST_ higher (Table 3). This pairwise *F*_ST_ pattern was highest for Kaʻena Point (average pairwise *F*_ST_ against all other populations = 0.173), which is consistent with the PCA in which Kaʻena Point individuals are highly differentiated from the six primarily recreational beaches. In our STRUCTURE analysis *K* = 2 was found to be the most likely number of clusters for our Oʻahu sampling (Supplementary Table S2). One cluster contains Kaʻena Point and Kaʻena North along with a few individuals from the Keawaʻula section of Kaʻena Point (Fig. 3). The other cluster contained the other half of Keawaʻula individuals, as well as the remaining populations. We explored the extent of additional sub-structuring in our dataset by analysing the results of K = 3 and K = 4 (Supplementary Figure S1). The *K* = 3 plot separates out eastern and western recreational beaches, while maintaining the populations at Kaʻena Point and Kaʻena North as being relatively distinct. Interestingly, at K = 4 Kaʻena Point and Kaʻena North begin to show distinct cluster membership.

**Table 3:** Pairwise *F*_ST_ between all combinations of populations sampled on Oʻahu.

	Kailua (KB)	Keawaʻula (KS)	Kaʻena North (KN)	Laniakea (LK)	Kaʻena Point (KP)	Mokuluēʻia (ML)	Makapuʻu (MP)
Keawaʻula (KS)	0.064						
Kaʻena North (KN)	0.125	0.108					
Laniakea (LK)	0.024	0.071	0.147				
Kaʻena Point (KP)	0.177	0.135	0.113	0.208			
Mokuluēʻia (ML)	0.075	0.087	0.147	0.082	0.204		
Makapuʻu (MP)	0.087	0.117	0.156	0.108	0.199	0.130	
Sandy’s (SB)	0.045	0.076	0.128	0.070	0.172	0.074	0.055

All pairwise comparisons were significant at *P* < 0.001.

### Estimating migration events

We used our population genomic dataset to then understand whether the populations on Oʻahu had any history of migrations. The optimum number of migration events and their placements varied depending on which block value (*k*) was used, with TreeMix supporting 1 (*k* = 75) to 2 (*k* = 100, 125) migration events. Some migration edges were detected in only a single block value analysis. For instance, the only migration event within *k* = 75 was detected going from Kaʻena Point to Keawaʻula (Supplementary Figure S2). Both *k* = 100 and *k* = 125 also had unique patterns with migration events of Makapuʻu going to Kailua Beach and Mokulēʻia going to Laniakea, respectively. Despite this variability, one migration event was consistently recovered across *k* = 100 and *k* = 125 analyses, which involved a migration even from Makapuʻu to the clade containing Kaʻena North and Kaʻena Point (Supplementary Figures S3 and S4). These results, along with others in our study, provide evidence of strong genetic admixture between populations on Oʻahu.

### Geographic barriers to gene flow and effective population size

Since the TreeMix analysis provided support for some migration, we further analysed these patterns via a Mantel test. Isolation by distance (IBD) was detected in our dataset, where we found a significant positive correlation between the genetic and geographic distance matrices in our dataset of eight populations (*r* = 0.429; *P* = 0.039). We found that effective population sizes (*N*_e_) ranged from 6.2 in Keawaʻula to 32 in Sandy’s Beach (Supplementary Table S2). While Kaʻena Point also had a relatively low *N*_e_ of 14.9, the trend was not universal for West Oʻahu as Kaʻena North had a *N*_e_ of 26.3.

### *F*_ST_ outlier analysis

To further dissect the contrast between the Kaʻena Point and Kaʻena North populations compared to the remaining six populations, we performed *F*_ST_ outlier analyses. Among the 923 SNPs tested, we found four SNPs to be significantly and consistently highly differentiated in five runs of the outlier detection programme BayeScan. The four outlier loci were found throughout the genome (Table 4). Three of the outlier SNPs were found in putative genes. However, these genes did not have an annotation that suggested a clear relationship to any observed phenotypic variation among these populations. Of the four outliers, three showed a pattern in which the Kaʻena Point vs. Sandy’s Beach was elevated (e.g. *F*_ST_ > 0.3; Table 4).

**Table 4:** BLAST results for *F*_ST_ outlier SNPs.

Chr.	SNP position^a^	*F*_ST_ KP vs. SB^b^	BLAST description	Query coverage	%ID^c^	E-value
1	13435854	0.33317	PREDICTED: *Bidens hawaiensis* kinesin-like protein KIN-UA (LOC143583003), misc_RNA	0.34	88.04	5E−54
5	24438623	0.408	PREDICTED: *Helianthus annuus* AAA-ATPase At4g25835 (LOC110893996), mRNA	0.11	78.7	4E−10
6	108010660	0.32075	PREDICTED: *Cynara cardunculus* var. *scolymus* protein SPIRRIG-like (LOC112505829), transcript variant X2, mRNA	0.87	79.95	2E−176
8	56539783	N/A^d^	No match			

^a^Position based on reference genome for *Scaevola taccada* (He et al. 2022, Li et al. 2023).

^b^Kaʻena Point versus Sandy’s Beach.

^c^Percent identity between search and query best hit.

^d^Kaʻena Point and Sandy’s Beach were fixed for the same allele and thus *F*_ST_ could not be calculated.

## Discussion

Our work shows that the ubiquitous dune shrub, *Scaevola taccada*, on the island of Oʻahu harbours significant genetic diversity and shows evidence of population genetic structuring. Paired with the leaf thickness trait analysis, we have shown that a mixture of genetic and phenotypic trait variation exists across a relatively small island. The documented diversity has important ramifications for the ability of the species to persist under the threat of climate change and sea level rise. The genetic structure of natural populations further indicates protected natural areas harbour distinct, but not more, genetic variation.

Genetic diversity is a prerequisite for adaptation to changing environments. Two populations that are subjected to less human activity, Kaʻena Point and Kaʻena North, both have some of the lowest values of genetic diversity, although not statistically different from all populations that experience more human activity. In general, the presence of genetic diversity within populations suggests that there is in fact a history of sexual reproduction despite their capacity for clonal propagation. It could be that the relatively lower genetic diversity in Kaʻena North/Point represents a mild founder effect or bottleneck. While Kaʻena Point has a lower effective population size, Kaʻena North is higher, thus indicating that any potential bottleneck in the area did not impact both populations equally. Furthermore, the levels of genetic diversity seen throughout Oʻahu suggest that *S. taccada* populations have not experienced a recent bottleneck due to habitat destruction as a result of human activities and/or climate change. While survival in changing environments can be driven by the levels of genetic diversity that can fuel local adaptation, phenotypic plasticity can also buffer species from environmental change. For example, in a common garden experiment on *Spartina densiflora* Brongn., populations had low genetic diversity, yet still exhibited significant phenotypic variation and plasticity for a number of traits (Castillo et al. 2018). Understanding the extent of local adaptation and the ability for phenotypic plasticity in *S. taccada* would require similar common garden-based studies.

We detected significant variation in leaf thickness across populations, but whether these differences are driven by genetic differentiation or environmental conditions is unclear. A future common garden study that eliminates environmental variation would be key to assessing potential genetical differentiation in this phenotype among populations. While the small number of populations precluded a sufficiently powered statistical analysis to detect correlations between leaf thickness and precipitation or temperature, geographically close populations like Kailua Beach and Makapuʻu that were genetically similar (Fig. 3) had significant differences in leaf thickness (Fig. 1d). The Kailua Beach and Makapuʻu populations also vary in average annual precipitation, with 708 mm of rain at Makapuʻu and 893 mm at Kailua Beach (Table 1). Prior work has highlighted the association of functional traits, like leaf succulence, and environmental conditions of *Scaevola* in Hawaiʻi (McKown et al. 2016). In *S. taccada*, increased salt exposure led to changes in leaf mass per area (LMA), but not in changes to leaf water content or carbon assimilation rates (Goldstein et al. 1996). A separate study showed increasing δ^13^ values with increased salt exposure (Alpha et al. 1996), suggesting increased water use efficiency. Together the prior work on *S. taccada* suggests investment in leaf mass (or thickness) may be a means by which to limit water loss by decreasing leaf hydraulic conductance, while we did not quantify leaf sap osmolarity of our leaf samples, sea spray and/or inundation variation across populations may be contributing to the significant variation in leaf thickness measured.

Populations on Oʻahu were significantly structured as determined by pairwise *F*_ST_, STRUCTURE, and PCA. All three methods produced concordant results in which the Kaʻena Point and Kaʻena North populations were significantly different from the other six populations. These populations are more protected (Kaʻena Point is a Natural Area Reserve and Kaʻena North is a State Park) and have larger geographic areas than five of the other populations. The Kaʻena Point area has a long history of intensive human use in the past 200 years (Wahl et al. 2022), including as pasture for livestock, railroads for the movement of sugarcane, and serving as a military complex. It was only in 1984 that the State Park was established (see the extensive history and documentation of Kaʻena Point found in Wahl et al. 2022). Interestingly, Kaʻena Point has been the focus of restoration activities in the past few decades, including planting naupaka (Susan Ching personal communication). Observations in the area suggest that Kaʻena Point might be a mixture of the plants from these restoration efforts as well as the ‘original’ population that preceded restoration. The population at Keawaʻula is geographically close to Kaʻena Point and Kaʻena North (being separated by rocky steep terrain) and spans between the protected population cluster (Kaʻena North/Point) and the other five non-protected populations on PC axis 1 (Fig. 2). Mokulēʻia is also relatively close to Kaʻena Point and Kaʻena North but shows little genetic similarity on PC1 (Fig. 2). These results together indicate that geography alone does not explain the patterns of genetic structure. Indeed, when we analysed our dataset for the presence of migration, we found evidence at both a small scale (from Kaʻena Point to Keawaʻula) and a wider scale (from Makapuʻu to Kaʻena Point and Kaʻena North) (Supplementary Figures S2–S4). These migration events further support the idea that while geography is clearly affecting population genetic structure in our dataset, populations across Oʻahu are not completely isolated.

In a species with an ocean-dispersing fruit, the ocean currents around the island could explain situations in which the geography does not mirror the population genetic structure. Typically, tradewinds blow out of the northeast towards the southwest when they encounter the Hawaiian islands. These winds impact ocean currents and by extension could dictate seed dispersal. For example, in Hawaiian fish (Counsell et al. 2021) and coral larvae (Storlazzi et al. 2006) currents play a notable role in dispersal dynamics. Because ocean currents are dynamic, change seasonally, and can be disproportionately impacted by large storm systems moving through the area, we are unable to infer that current-based dispersal is responsible for the structure we observe here. Since other nearby islands were not sampled, it is entirely possible that the different genetic clusters shown here could relate to ancestry from different Hawaiian islands. Finally, it should be noted that *S. taccada* is grown as an ornamental plant throughout the island. While we took great care to avoid sampling ornamental plants in yards/hotels, we cannot rule out the possibility that natural populations are undergoing gene flow, not only with other natural populations, but also the ornamental types. Future work that includes substantial ornamental plant samples would clarify if this is an important force shaping diversity on the island.

There have been a few other studies examining intraspecific genetic diversity of Hawaiian plants across Oʻahu. An endemic Hawaiian cotton, Maʻo or *Gossypium tomentosum* Nutt. ex Seem., was studied throughout the Hawaiian archipelago and across multiple sites on Oʻahu including Makapuʻu and near Kaʻena Point (DeJoode and Wendel 1992). This study used allozymes to assay genetic variation among few accessions from different locations, but did not sample at the population level and their results are not comparable to our study. However, the authors note that a PCA of their allozyme dataset indicated no geographic relationship with genetic diversity, whereas we saw differentiation within our dataset between those two similar sampling areas, which in turn suggests that the geographic relationships we show here are indeed species specific as opposed to being a more universal biogeographic pattern. A more recent study on a different Hawaiian endemic species, ʻŌhai or *Sesbania tomentosa* Hook. & Arn., used microsatellite markers to look at genetic diversity across the archipelago including populations in Kaʻena Point, Kaʻena North, as well as an island population just off the coast of Makapuʻu (Cole and Morden 2021). A STRUCTURE analysis showed that these Oʻahu populations clustered together, indicating an absence of genetic differentiation across Oʻahu. A study on ʻilima or *Sida fallax* Walp. also shared a similar Oʻahu sampling scheme (Pejhanmehr et al. 2024) as our study. Populations sampled from Makapuʻu and Kaʻena Point harboured lower levels of observed heterozygosity (0.1) than what we showed at those locations in this study (0.23–0.25; Table 2). The lower genetic diversity in ʻilima was paired with high genetic differentiation between Makapuʻu and Kaʻena Point (*F*_ST_ of approximately 0.2), which could reflect differences in pollination and/or seed dispersal (Pejhanmehr et al. 2024). *Scaevola taccada* populations from the southern islands of Japan (Ryukyu, Daito, and Ogasawara) were analysed to determine levels of gene flow within and among islands (Emura et al. 2022). While the study’s goal involved analysing a fruit dimorphism, their ddRAD-seq data still provides an interesting comparison point to our study. Genetic differentiation among populations (in their case measured as *F*_ST_^R^) was relatively similar to our findings in Oʻahu. The average within island *F*_ST_ in Japan *S. taccada* was approximately 0.08 compared to 0.07 on Oʻahu [this study].

Our analysis of *F*_ST_ outliers suggests that adaptation is not a predominant force in shaping genetic diversity in *S. taccada* on Oʻahu. Only three out of 923 SNPs supported the large differentiation we see in our PCA and STRUCTURE analysis between Kaʻena Point and east Oʻahu populations like Sandy’s Beach. It is possible that we missed important loci under selection due to the relatively low density of markers in this study. Furthermore, any outlier SNP is almost certainly linked to a casual variant as opposed to being in the relevant adaptive gene. The marked absence of a clear signal of rampant selection in this dataset suggests that the partitioning of genetic diversity among these populations may be primarily driven by neutral processes like gene flow, isolation, and genetic drift.

## Conclusions

In total, this work has highlighted both the genetic diversity and structure of natural populations of *S. taccada* on Oʻahu. While there was genetic differentiation, we determined that selection was not the primary driver of this differentiation. Instead, it is likely that isolation and gene flow are primarily shaping the distribution of genetic diversity. The fact that protected areas harboured distinct, but less, genetic diversity, shows that population dynamics on a relatively small island can be quite complex. It could suggest that the protected nature of those locations is discouraging terrestrial movement of propagules, thus building up the relatively modest levels of differentiation seen here. Alternatively, these patterns of population structure could be significantly impacted by dispersal patterns from other Hawaiian islands not sampled in this work. This work shows that on a single densely populated island in the Pacific Ocean that population genomics can reveal genetic differentiation between high human use and protected areas.

## Supplementary Material

plag042_Supplementary_Data

## Data Availability

All sequence data used in this study can be found in NCBI’s Short Read Archive under BioProject PRJNA1268958. The raw phenotyping data can be found in Supplementary Appendix S1.
