# Supplementary material for: Island-wide population genomic analysis of the coastal shrub, beach naupaka (Scaevola taccada L.), on Oʻahu
Source: AoB Plants. 2026 Sep 4;18(5):plag042. doi: 10.1093/aobpla/plag042 (PMC13584646; doi:10.1093/aobpla/plag042)
Supplement: Supplementary file 1 [file plag042_supplementary_data.zip › Supporting_Information.docx]

Supporting Information

Table S1: STRUCTURE Delta K analysis.

Table S2: Effective population size based on linkage disequilibrium.

Fig. S1: STRUCTURE plots.

Fig. S2: TreeMix analysis (k = 75).

Fig. S3: TreeMix analysis (k = 100).

Fig. S4: TreeMix analysis (k = 125).

**Table S1 -** STRUCTURE Delta K analysis for K=1 through K=9

| K | Reps | Mean LnP(K) | Stdev LnP(K) | Ln'(K) | \|Ln''(K)\| | Delta K |
| --- | --- | --- | --- | --- | --- | --- |
| 1 | 20 | -93142.24 | 0.4122 | NA | NA | NA |
| 2 | 20 | -88071.175 | 0.8188 | 5071.065 | 3271.64 | 3995.76751 |
| 3 | 20 | -86271.75 | 294.1001 | 1799.425 | 623.11 | 2.1187 |
| 4 | 20 | -85095.435 | 320.6479 | 1176.315 | 742.035 | 2.314174 |
| 5 | 20 | -84661.155 | 617.7936 | 434.28 | 336.11 | 0.544049 |
| 6 | 20 | -84562.985 | 147.1758 | 98.17 | 217.88 | 1.480407 |
| 7 | 20 | -84682.695 | 74.5107 | -119.71 | 130.055 | 1.745453 |
| 8 | 20 | -84672.35 | 73.8836 | 10.345 | 15.82 | 0.214121 |
| 9 | 20 | -84646.185 | 92.7565 | 26.165 | NA | NA |

Summary table showing the results of our STRUCTURE analysis in which we ran our SNP dataset through 20 replications at K=1 through K=9 [(Evanno *et al.* 2005)](https://paperpile.com/c/9GzX2I/JNTkT).

**Table S2 -** Effective population size (Ne) based on linkage disequilibrium patterns between SNPs

| Population | | N^1^ | Ne^2^ | 95CI_min | 95CI_max |
| --- | --- | --- | --- | --- | --- |
| SB | 19 | | 32 | 30.7 | 33.4 |
| KB | 9 | | 20 | 18.6 | 21.7 |
| KN | 20 | | 26.3 | 25.2 | 27.4 |
| KS | 9 | | 6.2 | 5.8 | 6.5 |
| ML | 19 | | 18.5 | 17.9 | 19.2 |
| MP | 15 | | 27.7 | 26.0 | 29.6 |
| KP | 17 | | 14.9 | 14.4 | 15.6 |
| LK | 5 | | N/A | N/A | N/A |

^1^ - Number of individuals sampled, ^2^ - Effective population size

**Supplementary Figure 1 –** STRUCTURE plots for additional values of K

Additional values of K (K=3 above; K=4 below) were plotted to explore genetic structure.

Alt text: STRUCTURE plots for three and four clusters. They show additional structure to the dataset that may have been obscured when focusing on just two clusters.

**Supplementary Figure 2.** TreeMix analysis (k = 75) using eight populations of *Scaevola taccada*: Kaʻena North (KN), Kaʻena Point (KP), Keawaʻula section of Kaʻena Point (KS), Kailua Beach (KB), Laniakea (LK), Makapuʻu (MP), Mokulēʻia (ML), Sandy’s (SB). The network includes three migration edges (m = 1) as identified by OptM. Branch lengths are proportional to the amount of genetic drift. Migration events are shown as arrows, with color intensity indicating migration weight as indicated by the color legend on the left.

Alt text: A treemix graphical display showing a phylogeny like structure with an arrow suggesting that Kaʻena Point population has some evidence of migration to Keawaʻula.

**Supplementary Figure 3.** TreeMix analysis (k = 100) using eight populations of *Scaevola taccada*: Kaʻena North (KN), Kaʻena Point (KP), Keawaʻula section of Kaʻena Point (KS), Kailua Beach (KB), Laniakea (LK), Makapuʻu (MP), Mokulēʻia (ML), Sandy’s (SB). The network includes four migration edges (m = 2) as identified by OptM. Branch lengths are proportional to the amount of genetic drift. Migration events are shown as arrows, with color intensity indicating migration weight as indicated by the color legend on the left.

Alt text: A treemix graphical display showing a phylogeny like structure with two arrows suggesting that the population of Makapuʻu has some evidence of migration to Kailua Beach and Kaʻena North.

**Supplementary Figure 4.** TreeMix analysis (k = 125) using eight populations of *Scaevola taccada*: Kaʻena North (KN), Kaʻena Point (KP), Keawaʻula section of Kaʻena Point (KS), Kailua Beach (KB), Laniakea (LK), Makapuʻu (MP), Mokulēʻia (ML), Sandy’s (SB). The network includes two migration edges (m = 2) as identified by OptM. Branch lengths are proportional to the amount of genetic drift. Migration events are shown as arrows, with color intensity indicating migration weight as indicated by the color legend on the left.

Alt text: A treemix graphical display showing a phylogeny like structure with two arrows suggesting that the population of Mokulēʻia has migration evidence to Laniakea and Makapuʻu has migration evidence to Kaʻena.
